# Holter-Derived Autonomic Function, Arrhythmias and Carbohydrate Metabolism in Patients with Class III Obesity Treated with Laparoscopic Sleeve Gastrectomy

**DOI:** 10.3390/jcm10102140

**Published:** 2021-05-15

**Authors:** Piotr Bienias, Zuzanna Rymarczyk, Justyna Domienik-Karłowicz, Wojciech Lisik, Piotr Sobieraj, Piotr Pruszczyk, Michał Ciurzyński

**Affiliations:** 1 Department of Internal Medicine and Cardiology, Medical University of Warsaw, 02-005 Warsaw, Poland; zmrymarczyk@gmail.com (Z.R.); jdomienik@o2.pl (J.D.-K.); piotr.pruszczyk@wum.edu.pl (P.P.); michal.ciurzynski@wum.edu.pl (M.C.); 2 Department of General Surgery and Transplantology, Medical University of Warsaw, 02-005 Warsaw, Poland; wojciech.lisik@wum.edu.pl; 3 Department of Internal Medicine, Hypertension and Vascular Diseases, Medical University of Warsaw, 02-005 Warsaw, Poland; piotr.sobieraj@wum.edu.pl

**Keywords:** class III obesity, cardiac autonomic function, heart rate variability, arrhythmias, insulin resistance

## Abstract

The effects of weight loss following bariatric surgery on autonomic balance, arrhythmias and insulin resistance are still of interest. We prospectively investigated 50 patients with BMI > 40 kg/m^2^, aged 36.5 (18–56) years who underwent laparoscopic sleeve gastrectomy. Among other examinations, all subjects had 24-h Holter monitoring with heart rate variability (HRV) and heart rate turbulence (HRT) evaluation. After a median of 15 months, BMI decreased from 43.9 to 29.7 kg/m^2^, the incidence of hypertension decreased from 54 to 32% (*p* = 0.04) and any carbohydrate disorders decreased from 24 to 6% (*p* = 0.02). Fasting insulin concentration and insulin resistance index improved significantly (*p* < 0.001). Improvements in HRV parameters related to the sympathetic autonomic division were also observed (*p* < 0.001), while HRT evaluation was not conclusive. The enhancement of autonomic tone indices was correlated with reduction of BMI (SDNN-I r = 0.281 *p* = 0.04; SDNN r = 0.267 *p* = 0.05), but not with reduction of waist circumference, and it was also associated with decrease of mean heart rate (OR 0.02, 95%CI 0.0–0.1, *p* < 0.001). The incidence of arrhythmias was low and similar before and after follow-up. In conclusion, improvement of homeostasis of carbohydrate metabolism and autonomic function is observed in relatively young patients after weight loss due to laparoscopic sleeve gastrectomy.

## 1. Introduction

Morbid obesity may cause various cardiovascular complications, including hypertension, arrhythmias and cardiac autonomic nervous system (ANS) abnormality. Obesity is also associated with dysfunctional metabolic status including a higher incidence of various carbohydrate metabolism disorders and insulin resistance. In addition, some results suggest an association between hyperinsulinemia and insulin resistance in obesity and ANS imbalance [1,2,3].

Cardiac complications in obesity can be complex and result from left ventricular systolic or diastolic dysfunction, atrial dilatation and its electrical remodeling, myocyte hypertrophy, fibrosis and fatty infiltration. Many studies, including meta-analyses, suggest that weight loss after bariatric surgery is associated with significant improvements in cardiac morphology and function [1,4]. The effects of weight loss following various techniques of bariatric surgery on hyperinsulinemia, insulin resistance and obesity associated neuropathy are still of interest. Available data suggest that various techniques of bariatric surgery improve metabolic control, including glycemic homeostasis and increased insulin sensitivity [5,6,7,8,9]. However, limited recent results are available on assessment of the dynamics of fasting glucose, insulin levels, insulin resistance/sensitivity indices and also ANS indices in patients with class III obesity, who lost weight due to laparoscopic sleeve gastrectomy (LSG), especially at a relatively young age and without severe accompanying diseases. The available data often provide inconclusive and contradictory results too [7,10,11,12,13]. There is also no detailed evaluation of the impact of weight loss following bariatric surgery on the occurrence of various arrhythmias [14,15].

Therefore, we assessed patients with class III obesity in detail before LSG and after 12–18 months of follow-up. Our aim was to investigate the influence of weight loss on cardiac autonomic function and arrhythmias (primary endpoint) as well as selected parameters of carbohydrate metabolism (secondary endpoint) in relation to anthropometric measurements of obesity. Next to laboratory tests, we focused on detailed evaluation of Holter-derived time-domain heart rate variability (HRV) and heart rate turbulence (HRT), as they are established methods for assessing cardiac ANS function [16,17,18]. Both insulin resistance and HRV and HRT are also independent and important predictors of future cardiac, neurological and metabolic health. Other studies mainly assessed the metabolic status and ANS early after bariatric surgery; thus, we decided to check these conditions later, i.e., >12 months after surgery. Our research hypothesis was as follows: decreases in body mass index (BMI) and waist circumference (WC) after LSG due to class III obesity resulted in multi-profile improvements in cardiac ANS function, heart rhythm disturbances and carbohydrate metabolism.

## 2. Material and Methods

### 2.1. Study Population and Laboratory Tests

This is a single center, prospective cohort study. Fifty adult patients aged ≥18 years with initially BMI ≥ 40 kg/m^2^ who underwent LSG were selected for the study evaluation. Patients were examined at the start of the study and after 12–18 months of follow-up. Our group was drawn from 81 individuals with class III obesity who were referred to LSG, as detailed in our previous publication (including also standard 12-lead electrocardiography and echocardiography) [19]. In this report, we present 50 patients who underwent LSG and underwent follow-up examination. Others were not finally qualified for surgery or did not report for a follow-up visit within the required period.

All subjects were stable outpatients who underwent 24-h Holter monitoring and basic laboratory tests, including insulin levels. On the basis of fasting glucose and insulin levels, the insulin resistance index (HOMA-IR, homeostatic model assessment to quantify insulin resistance, normal value ≤0.9) and also the insulin sensitivity index (QUICKI, Quantitative Insulin Sensitivity Check Index, normal value ≥0.34) were calculated according to widely available specific formulas.

To avoid the influence of various factors that are well known to strongly affect both cardiac ANS and arrhythmias, we did not include patients with various clinical or laboratory abnormalities, i.e., chronic coronary syndromes, heart failure with reduced ejection fraction <50%, significant heart valvular abnormalities, poorly controlled arterial hypertension, earlier confirmed by polysomnography obstructive sleep apnea syndrome, unexplained anemia (hemoglobin <12.0 g/l), uncontrolled thyroid dysfunction and reduction in glomerular filtration rate <60 mL/min, according to the Cockcroft–Gault equation. Since the assessment of cardiac ANS function based on Holter recording is possible only in people with sinus rhythm, and patients with persistent or permanent atrial fibrillation or flutter were also excluded. Studied patients cannot use anti-arrhythmic drugs class I–IV according to Vaughan Williams classification for any reasons (including beta-blockers or non-dihydropyridine calcium antagonists). Use of ≥2 antihypertensive medications at full doses was also an exclusion criterion. Patients with other acute or significant chronic diseases were not included either. All patients gave their written informed consent to participate in the study. This study was conducted in accordance with the amended Declaration of Helsinki. The protocol of the study was accepted by the Bioethics Committee of the Medical University of Warsaw, Poland (protocol no. AKBE/108/15).

### 2.2. 24-h Holter Monitoring

The Holter monitoring was recorded during normal everyday activity on a 3-channel digital device (Lifecard CF, Spacelabs Healthcare, Snoqualmie, WA, USA). An evaluation of heart rate, various arrhythmias and time-domain HRV was performed (Sentinel Impresario, Spacelabs Healthcare, WA, USA). According to European and American Task Force 6, indices of time-domain HRV were measured (full names and abbreviations in Table 1) [20]. The SDNN and HRV-Index estimated of overall HRV, SDANN estimated of long-term components of HRV and RMSSD and pNN50 estimated of short-term components of HRV. Two numerical HRT parameters after ventricular extrasystoles, e.g., turbulence onset and turbulence slope, were calculated using custom designed software based on the described methodology (details in our previous article) [19,21]. All HRV and HRT parameters were evaluated for the full 24 h without separation for the day and night periods. Holter recording was analyzed by the qualified cardiologist. 

### 2.3. Statistical Analysis

The tested groups were compared by either Student’s *t*-test or the Mann–Whitney–Wilcoxon test, according to parameters’ distribution assessed by the Shapiro–Wilk test (variables with normal distribution were presented as mean with standard deviation, not showing normal distribution as median with range values). Deletions of outliers’ data were not performed. The χ2 test or McNemar’s test was used to compare categorical variables (if needed, Yates’s correction was applied). All tests were double-sided. Correlations were evaluated by Spearman correlation coefficients. Logistic regression analysis was carried out to explore the influence of confounding factors on cardiac autonomic function in patients with obesity. The influence of measured parameters was expressed as an odds ratio (OR) with 95% confidence interval (CI). Values of *p* < 0.05 were considered statistically significant. Analyses were performed using R, which is a free software environment for statistical computing and graphics (www.r-project.org, version 3.4.0, accessed on 1st May 2017).

## 3. Results

### 3.1. Clinical Characteristics of Study Populations

**Figure 1:**
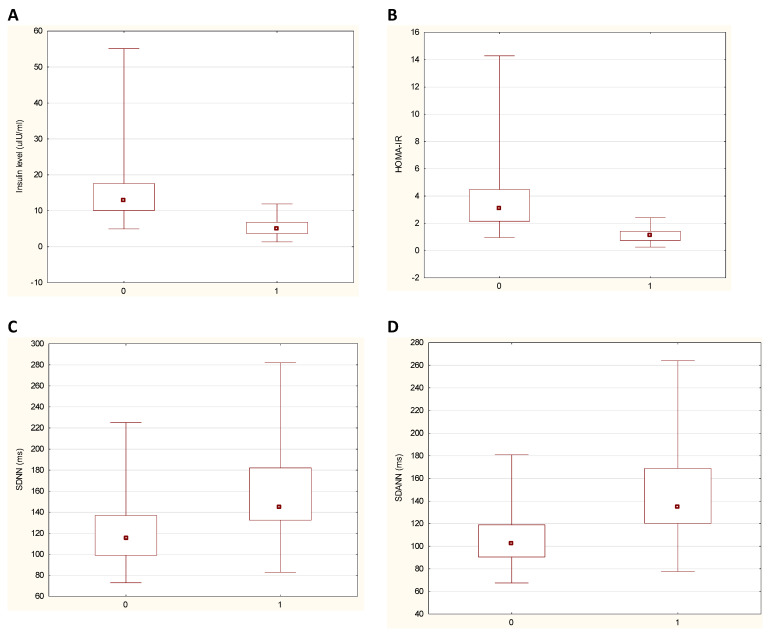
The values of fasting insulin level (chart **A**), homeostatic model assessment for insulin resistance HOMA-IR (chart **B**), SDNN (chart **C**) and SDANN (chart **D**) in 50 patients before and after weight loss. Charts present medians with ranges’ values, while detailed results are shown in Table 2 (*Y*-axis: 0—patients before bariatric surgery, 0—patients after weight loss).

The general characteristics of the patients with obesity before and after weight loss are presented in Table 2. The median age was 36.5 years (range 18–56), and 86% of the study cohort were women. It is worth noting that after observation, the incidence of carbohydrate metabolism disorders and hypertension decreased significantly, and all laboratory tests improved—results in Table 2 and in Figure 1. During the follow-up visit, patients received previously started angiotensin converting enzyme inhibitors (in 16/32%), diuretics (in 6/12%), dihydropyridine calcium antagonists (in 5/10%) and also a statin or fibrate (in 18/36%) due to primary prevention. One patient with type 2 diabetes mellitus was taking insulin, while two patients with persistent impaired glucose tolerance were receiving metformin. None of the subjects received beta-blockers or other medications that affected heart rhythm. 

### 3.2. 24-h Holter Data

Detailed results of 24-h Holter data before and after weight loss are presented in Table 1. After observation, a significant improvement was observed in HRV indices estimating overall and long-term components, which are mainly related to the sympathetic tone (SDNN, SDANN and HRV-Index). By contrast, RMSSD and pNN50 values estimating short-term components and mainly associated with parasympathetic regulation remained unchanged. Due to rare occurrences of ventricular extrasystoles, HRT parameters were possible to count in only 10 subjects both before as well as after bariatric surgery. In the obtained results, turbulence slope (mostly triggered by a sympathetic tone) and also turbulence onset value (mostly related to transient vagal inhibition) were not significantly changed after weight loss. 

**Figure 2:**
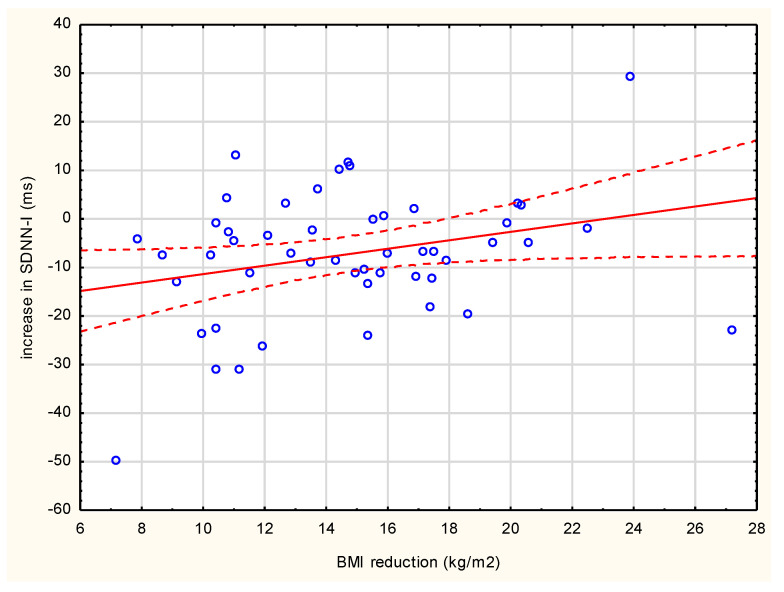
Correlation between the reduction in body mass index and the increase in SDNN-I value (r = 0.281, *p* = 0.04) in all 50 patients after follow-up period.

Correlations were measured to estimate the association between a reduction in BMI or a reduction in WC and an increase in HRV indices. There were significant correlations between BMI reduction and increase of SDNN-I (r = 0.281, *p* = 0.04; Figure 2) and also nearly significant correlations for increase of SDNN (r = 0.267, *p* = 0.05) and SDANN (r = 0.256, *p* = 0.07). However, no correlations between the increase of HRV and WC reduction or HOMA-IR reduction were observed in patients after follow-up. 

In addition, no significant differences in parameters of carbohydrate metabolism, HRV and HRT were observed after follow-up in patients divided according to the degree of weight reduction expressed by median of final BMI or final WC—results in Table 3. There were also no significant differences in the improvement of patients’ HRV parameters according to the median follow-up period (<15 vs. ≥15 months)—detailed data are not shown. 

The univariate logistic regression analysis was performed to detect potential predictors of increase of SDNN after weight loss (the main HRV parameter). This analysis revealed that only mean heart rate was significantly related to the increase in SDNN value (odds ratio 0.02, 95%CI 0.0–0.1, *p* < 0.001). Other parameters used in the univariate analysis included age, BMI reduction and WC reduction (the detailed values of the corrections applied are not presented). Due to the results of the univariate analysis, the previously planned multivariate analysis was not performed.

## 4. Discussion

Obesity is a multi-factorial disease, and obesity-related diseases increase the incidence of disability and mortality [1,2,22]. The main finding of our study is that the weight loss after LSG resulted in a multi-profile improvement in carbohydrate metabolism and blood pressure control, as well as overall cardiac ANS function. In addition, these health benefits were observed irrespective of degree of weight loss. 

Various techniques of bariatric surgery are used for effective treatment of morbid obesity, such as Roux-en-Y gastric bypass, sleeve gastrectomy or biliopancreatic diversion with duodenal switch. In most patients, all types of bariatric surgery procedures improve metabolic status, reduce the incidence of hypertension and decrease long-term mortality [2,22]. Multiple studies and meta-analyses suggest that weight loss following bariatric surgery is associated with significant optimization of glycemia, insulin, lipids and other metabolic and hormonal changes that improve the overall metabolic profile [2,9,22]. Several hypotheses have been put forward trying to explain individually variable improvement, and one of the issues studied is the role of the ANS function in this process [13,23]. However, these mechanisms are extremely complex and still not fully understood [2,7,11,24].

Recently, one of the preferred procedures is LSG with relatively few postoperative complications. As numerous studies have shown, ANS dysfunction is often found because of morbid obesity, while weight loss improves sympathetic and parasympathetic activity and consequently decreases mean heart rate [6,10,13,25]. After analyzing the results of previous studies, we expected not only HRV and HRT recovery, but also a reduction in the incidence of hypertension and improvement in glucose homeostasis. Our study confirmed these assumptions, and we additionally showed that positive changes are present regardless of the degree of weight loss expressed by the median reduction of BMI or WC (Table 3). It has also been hypothesized that changes in the vagal-modulated neuroendocrine system have an additional effect on the beneficial effects after bariatric surgery [6,10,13,25]. Both the Roux-en-Y surgery and sleeve gastrectomy may induce metabolic improvements via different mechanisms. In a recent Greek study, both these surgical procedures resulted in comparable improvements in glucose, HOMA-IR, triglycerides and high-density lipoprotein cholesterol, while insulin levels were significantly higher in the sleeve gastrectomy group [9]. Earlier observations have suggested that LSG has more benefits in improving autonomic balance because the vagus nerve fibers are not damaged during this procedure, in contrast to the Roux-en-Y method, where induced damage to the vagus nerve innervation is similar to that of a sub-diaphragmatic trunk vagotomy [10,26,27]. However, in our study, HRV indices related to parasympathetic part did not improve after the follow-up period. Thus, our evaluation does not support the hypothesis that LSG causes significant improvements in both parts of ANS function.

Another issue concerns the relationship between anthropometric parameters and autonomic HRV indices. It seems that improvement of sympathetic parameters should be related to the decrease of WC, as abdominal obesity is just associated with hyperinsulinemia, hyperleptinemia and insulin resistance, which are considered to be contributed to the abnormal activation of the sympathetic autonomic system [28]. However, in our study, correlations between an increase of SDNN, SDNN-I and SDANN, and a reduction in BMI were revealed, but not with the reduction in waist circumference. 

The results of the study by Sharma et al. suggest that sleeve gastrectomy leads to a dramatic improvement in insulin resistance as early as the first postoperative day [29]. In our study, HOMA-IR was also significantly lower after a median of 15 months of follow-up. Literature data on the association of HRV with insulin resistance parameters in patients after bariatric surgery are not consistent. As in the results of the study by Maser et al., no correlations between the increase of HRV parameters and HOMA-IR reduction were observed in our individuals after follow-up [12]. In contrast, an evaluation by Wu et al. revealed significant association between changes in HOMA-IR and increase of parasympathetic-related HRV indices 180 days after LSG [30]. Nevertheless, the Geronikolou et al. meta-analysis of 646 patients aged 34–52.5 years and BMI >50 kg/m^2^ showed a positive effect of weight loss after various bariatric surgeries not only on time-domain or frequency-domain HRV parameters, but also on HOMA-IR. Interestingly, the authors concluded that gastric bypass favors insulin resistance decrease, while sleeve gastrectomy increases the vagal tone. Accordingly, in patients with severe cardiovascular involvement, a sleeve gastrectomy should be preferred to gastric bypass techniques [27]. It is worth emphasizing that studies involving many cases indicate that next to insulin resistance improvement, remission of type 2 diabetes after bariatric surgery is also possible, especially from operations with a malabsorptive component [2].

Hitherto, the assessment of HRT was infrequently performed in patients with obesity or metabolic syndrome [31,32]. In our study, the small number of people with ventricular extrasystoles suitable to calculate HRT significantly limited the statistical analysis both before and after weight loss. As far as we know, such evaluation during follow-up has not been performed in patients treated with bariatric surgery as of yet; therefore, this promising issue requires further research. 

There is ample evidence that various bariatric procedures and subsequent weight loss significantly reduce the incidence of cardiovascular complications and improve the structure and function of the heart [4,15]. However, there are limited data evaluating the incidence of arrhythmias after bariatric surgery [14,15,33,34]. In the presented patients, it was surprising that numerous or severe cardiac arrhythmias were very rare, including atrial fibrillation or nocturnal bradyarrhythmia. However, our study included subjects with a median of 36.5 years, without structural heart disease and other serious comorbidities, including evident obstructive sleep apnea that might predispose to arrhythmias. In Holter monitoring, atrial tachycardia was even more common after follow-up, with the exception of atrial fibrillation. However, the prognostic value of frequent atrial tachycardia is limited, and so far, no clear association between these arrhythmias and cardiac ANS function has been demonstrated. It is noteworthy that after weight loss, short non-sustained ventricular tachycardia was also reported in two patients (both with slight left ventricular hypertrophy 10–11 mm of wall thickness recognized during at baseline visit).

One of the limitations of our study is the small number of patients enrolled, which undoubtedly influenced the obtained results, but many publications on morbid obesity are of similar size. In particular, the subgroup analysis (presented in Table 3) concerns a small number of compared patients; therefore, the differences may not be significant. An additional reason for the results obtained may be that our group was relatively young and consisted mainly of women. In addition, to eliminate the influence of various factors on examined parameters, only selected patients were included as described in the Methods section. Another possible limitation is the lack of frequency-domain (power spectral) HRV analysis. However, we are convinced that well-tried time-domain HRV and HRT analyses are sufficient for assessing ANS function. 

## 5. Conclusions

In our study, the weight loss after LSG due to class III obesity resulted in a multi-profile improvement in carbohydrate metabolism and blood pressure control as well as overall cardiac ANS function. The improvement of HRV sympathetic-related indices were correlated with the reduction of BMI, but not with the reduction of WC. The subgroup analysis according to the degree of reduction of anthropomorphic parameters (BMI, WC) suggested that health benefits after LSG might be expected even in people with less weight loss. Serious or life-threatening cardiac arrhythmia were infrequent both before surgical treatment as well as after weight loss. However, it should be noted that patients evaluated in our study were of relatively young age and without other significant comorbidities. 

## Figures and Tables

**Table 1:** Arrhythmias and heart rate variability parameters in 24-h Holter monitoring in patients before and after bariatric surgery.

Characteristic	Patients Before Bariatric Surgery (*n* = 50)	Patients After Bariatric Surgery (*n* = 50)	*p* Value
**Heart rate ^1^**			
Mean heart rate (bpm)	80 ± 11	74 ± 10	<0.001
Minimal heart rate (bpm) *	58 (39–89)	51 (33–68)	<0.001
Maximal heart rate (bpm)	127 (100–181)	126 (105–167)	0.21
**Supraventricular arrhythmias (no., %)**			
Supraventricular extrasystoles >100/24 h	2 (4%)	3 (6%)	1.0
Non-sustained supraventricular tachycardia ^2^	5 (10%)	15 (30%)	0.02
**Ventricular arrhythmias (no., %)**			
Ventricular extrasystoles >100/24 h	1 (2%)	3 (6%)	0.62
Non-sustained ventricular tachycardia ^2^	0 (0%)	2 (4%)	0.49
**Time-domain heart rate variability parameters ^3^**			
SDNN (ms) *	115 (73–225)	145 (83–282)	<0.001
SDNN-I (ms) *	41 (20–115)	45 (24–122)	<0.001
SDANN (ms) *	102 (68–181)	134 (78–264)	<0.001
RMSSD (ms) *	34 (15–122)	33 (16–112)	0.25
pNN50 (%) *	8.6 (0.3–44.1)	9.3 (0.4–50.8)	0.06
HRV-index *	16 (10–35)	22 (11–38)	<0.001
**Heart rate turbulence parameters ^4^**			
Turbulence onset (%) *	−2.1 (−7.5–−0.6)	−3.6 (−8.6–1.2)	0.73
Turbulence slope (ms/RR) *	7.5 (−3.1–21.8)	8.6 (−3.1–43.8)	0.22
Abnormal HRT (no.,%)	3 (30%)	2 (20%)	0.80

* Values presented as median with range. ^1^ All patients presented sinus rhythm. ^2^ Non-sustained tachycardia were recognized when the rate was >100 beats per minute for at least 3 consecutive beats and arrhythmia lasted <30 s. ^3^ SDNN—the standard deviation of N-N (normal-to-normal) interval; SDNN-I—is the mean of the standard deviations of all the NN intervals for each 5 min periods of the entire recording; SDANN—the standard deviation of the average of N-N in all 5 min periods of the entire recording; RMSSD—the square root of the mean of the sum of the squares of differences between adjacent N-N; pNN50—number of pairs of adjacent N-N differing by more than 50 ms in the entire recording divided by the total number of all N-N; HRV Index—total number of all NN intervals divided by the height of the histogram of all NN intervals measured on a discrete scale with bins of 1/128 s 23 [20]. ^4^ HRT values were possible to measure in only 10 obese patients before and after bariatric surgery; as proposed by International Society for Holter and Noninvasive Electrocardiology, abnormal HRT was recognized if TO value was ≥0% and/or TS value was ≤2.5 ms/RR [21].

**Table 2:** Anthropometric obesity parameters, additional diseases and parameters of carbohydrate metabolism results in patients before and after bariatric surgery.

Characteristic	Patients Before Bariatric Surgery (*n* = 50)	Patients After Bariatric Surgery (*n* = 50)	*p* Value
Body mass index (kg/m^2^) *	43.9 (40.1–55.8)	29.7 (19.6–43.9)	<0.001
Body mass index reduction (kg/m^2^) *	-	14.7 (7.2–23.9)	-
Waist circumference (cm) *	139 (127–155)	88 (67–124)	<0.001
Waist circumference reduction (cm) *	-	53 (18–74)	-
**Additional diseases and parameters of carbohydrate metabolism**			
Hypertension, *n* (%)	27 (54%)	16 (32%)	0.04
Disorders of carbohydrate metabolism (together), (*n*,%)	12 (24%)	3 (6%)	0.02
Type 2 diabetes mellitus, (*n*,%)	1 (2%)	1 (2%)	1.0
Impaired glucose tolerance, (*n*,%)	6 (12%)	2 (4%)	0.27
Impaired fasting glucose, (*n*,%)	5 (10%)	0 (0%)	0.05
Fasting glucose level (mg/dl) *	90 (76–118)	85 (64–98)	<0.001
Fasting insulin level (uIU/mL) *	13.0 (5.0–55.1)	5.0 (1.4–11.9)	<0.001
QUICKI *^,1^	0.32 (0.27–0.39)	0.38 (0.33–0.49)	<0.001
HOMA-IR *^,2^	3.1 (1.0–14.3)	1.1 (0.3–2.4)	<0.001

* Values presented as median with range. ^1^ QUICKI-quantitative insulin sensitivity check index. ^2^ HOMA-IR-homeostatic model assessment for insulin resistance.

**Table 3:** Comparison of indices of carbohydrate metabolism, time-domain heart rate variability and heart rate turbulence in groups divided according to the median reduction of body mass index and the median reduction of waist circumference.

Characteristic	Patients with Body Mass Index Reduction <14.7 kg/m^2^ (*n* = 24)	Patients with Body mass Index Reduction ≥14.7 kg/m^2^ (*n* = 26)	*p* value	Patients with Waist Circumference Reduction <53 cm (*n* = 24)	Patients with Waist Circumference Reduction ≥53 cm (*n* = 26)	*p* value
Hypertension, n (%)	10 (42%)	6 (23%)	0.23	8 (33%)	8 (31%)	1.0
**Parameters of carbohydrate metabolism**						
Fasting glucose level (mg/dl) *	86.6 ± 5.3	83.0 ± 6.8	0.05	85.7 ± 5.4	83.9 ± 7.0	0.33
Fasting insulin level (uIU/mL) *	5.5 ± 2.3	5.2 ± 2.4	0.69	5.7 ± 2.6	5.0 ± 2.0	0.32
QUICKI *^,1^	0.38 ± 0.03	0.39 ± 0.04	0.40	0.38 ± 0.03	0.39 ± 0.03	0.47
HOMA-IR * ^1^	1.17 ± 0.51	1.09 ± 0.52	0.54	1.21 ± 0.56	1.06 ± 0.46	0.28
**Time-domain heart rate variability parameters ^1^**						
SDNN (ms) *	156 (99–244)	138 (83–282)	0.15	143 (99–269)	143 (83-282)	0.56
SDNN-I (ms) *	54 (28–93)	44 (24–122)	0.23	49 (24–121)	44 (26–96)	0.78
SDANN (ms) *	144 (95–257)	129 (78–264)	0.25	133 (95–223)	133 (78–264)	0.36
RMSSD (ms) *	40 (19–90)	28 (16–112)	0.14	38 (16–112)	33 (18–108)	0.62
pNN50 (%) *	11.9 (1.1–37.8)	5.7 (0.4–50.8)	0.12	10.3 (0.4–50.8)	9.3 (0.8–40.9)	0.47
HRV–index *	23 (15–37)	21.8 (11–38)	0.38	27 (15–38)	29 (11–36)	0.26
**Heart rate turbulence parameters**						
Turbulence onset (%)	−2.9 ± 3.4	−3.1 ± 2.9	0.93	−3.5 ± 2.8	-2.7 ± 3.2	0.51
Turbulence slope (ms/RR)	12.6 ± 12.9	16.2 ± 14.2	0.58	17.3 ± 13.1	12.2 ± 13.8	0.62

* Values presented as median with range. ^1^ For abbreviations—see Table 2.

## Data Availability

Not applicable.

## References

[1] Dwivedi A.K., Dubey P., Cistola D.P., Reddy S.Y. (2020). Association Between Obesity and Cardiovascular Outcomes: Updated Evidence from Meta-analysis Studies. Curr. Cardiol. Rep..

[2] Keshavjee S.H., Schwenger K.J.P., Yadav J., Jackson T.D., Okrainec A., Allard J.P. (2021). Factors Affecting Metabolic Outcomes Post Bariatric Surgery: Role of Adipose Tissue. J. Clin. Med..

[3] Straznicky N.E., Grima M.T., Eikelis N., Nestel P.J., Dawood T., Schlaich M.P., Chopra R., Masuo K., Esler M.D., Sari C.I. (2011). The effects of weight loss versus weight loss maintenance on sympathetic nervous system activity and metabolic syndrome components. J. Clin. Endocrinol. Metab..

[4] Aggarwal R., Harling L., Efthimiou E., Darzi A., Athanasiou T., Ashrafian H. (2016). The Effects of Bariatric Surgery on Cardiac Structure and Function: A Systematic Review of Cardiac Imaging Outcomes. Obes. Surg..

[5] Azmi S., Ferdousi M., Liu Y., Adam S., Iqbal Z., Dhage S., Ponirakis G., Siahmansur T., Marshall A., Petropoulos I. (2021). Bariatric surgery leads to an improvement in small nerve fibre damage in subjects with obesity. Int. J. Obes..

[6] Ibacache P., Carcamo P., Miranda C., Bottinelli A., Guzman J., Martinez-Rosales E., Artero E.G., Cano-Cappellacci M. (2020). Improvements in Heart Rate Variability in Women with Obesity: Short-term Effects of Sleeve Gastrectomy. Obes. Surg..

[7] Gomide Braga T., das Gracas Coelho de Souza M., Maranhao P.A., Menezes M., Dellatorre-Teixeira L., Bouskela E., Le Roux C.W., Kraemer-Aguiar L.G. (2020). Evaluation of Heart Rate Variability and Endothelial Function 3 Months After Bariatric Surgery. Obes. Surg..

[8] Ashrafian H., Harling L., Toma T., Athanasiou C., Nikiteas N., Efthimiou E., Darzi A., Athanasiou T. (2016). Type 1 Diabetes Mellitus and Bariatric Surgery: A Systematic Review and Meta-Analysis. Obes. Surg..

[9] Magouliotis D.E., Tasiopoulou V.S., Sioka E., Chatedaki C., Zacharoulis D. (2017). Impact of Bariatric Surgery on Metabolic and Gut Microbiota Profile: A Systematic Review and Meta-analysis. Obes. Surg..

[10] Casellini C.M., Parson H.K., Hodges K., Edwards J.F., Lieb D.C., Wohlgemuth S.D., Vinik A.I. (2016). Bariatric Surgery Restores Cardiac and Sudomotor Autonomic C-Fiber Dysfunction towards Normal in Obese Subjects with Type 2 Diabetes. PLoS ONE.

[11] Cornejo-Pareja I., Clemente-Postigo M., Tinahones F.J. (2019). Metabolic and Endocrine Consequences of Bariatric Surgery. Front Endocrinol..

[12] Maser R.E., Lenhard M.J., Peters M.B., Irgau I., Wynn G.M. (2013). Effects of surgically induced weight loss by Roux-en-Y gastric bypass on cardiovascular autonomic nerve function. Surg. Obes. Relat. Dis..

[13] Perugini R.A., Li Y., Rosenthal L., Gallagher-Dorval K., Kelly J.J., Czerniach D.R. (2010). Reduced heart rate variability correlates with insulin resistance but not with measures of obesity in population undergoing laparoscopic Roux-en-Y gastric bypass. Surg. Obes. Relat. Dis..

[14] Clapp B., Amin M., Dodoo C., Harper B., Liggett E., Davis B. (2020). New Onset Cardiac Arrhythmias after Metabolic and Bariatric Surgery. JSLS.

[15] Lee G.K., Cha Y.M. (2016). Cardiovascular benefits of bariatric surgery. Trends Cardiovasc. Med..

[16] Cygankiewicz I. (2013). Heart rate turbulence. Prog. Cardiovasc. Dis..

[17] Cygankiewicz I., Zareba W. (2013). Heart rate variability. Handb. Clin. Neurol..

[18] Williams S.M., Eleftheriadou A., Alam U., Cuthbertson D.J., Wilding J.P.H. (2019). Cardiac Autonomic Neuropathy in Obesity, the Metabolic Syndrome and Prediabetes: A Narrative Review. Diabetes Ther..

[19] Bienias P., Rymarczyk Z., Domienik-Karlowicz J., Lisik W., Sobieraj P., Pruszczyk P., Ciurzynski M. (2021). Assessment of arrhythmias and cardiac autonomic tone at a relatively young age patients with obesity class III. Clin. Obes..

[20] (1996). Heart rate variability. Standards of measurement, physiological interpretation, and clinical use. Task Force of the European Society of Cardiology and the North American Society of Pacing and Electrophysiology. Eur. Heart J..

[21] Bauer A., Malik M., Schmidt G., Barthel P., Bonnemeier H., Cygankiewicz I., Guzik P., Lombardi F., Muller A., Oto A. (2008). Heart rate turbulence: Standards of measurement, physiological interpretation, and clinical use: International Society for Holter and Noninvasive Electrophysiology Consensus. J. Am. Coll. Cardiol..

[22] Cardoso L., Rodrigues D., Gomes L., Carrilho F. (2017). Short- and long-term mortality after bariatric surgery: A systematic review and meta-analysis. Diabetes Obes. Metab..

[23] Rossi R.C., Vanderlei L.C., Goncalves A.C., Vanderlei F.M., Bernardo A.F., Yamada K.M., da Silva N.T., de Abreu L.C. (2015). Impact of obesity on autonomic modulation, heart rate and blood pressure in obese young people. Auton. Neurosci..

[24] Rao R.S., Yanagisawa R., Kini S. (2012). Insulin resistance and bariatric surgery. Obes. Rev..

[25] Alam I., Lewis M.J., Lewis K.E., Stephens J.W., Baxter J.N. (2009). Influence of bariatric surgery on indices of cardiac autonomic control. Auton. Neurosci..

[26] Ballsmider L.A., Vaughn A.C., David M., Hajnal A., Di Lorenzo P.M., Czaja K. (2015). Sleeve gastrectomy and Roux-en-Y gastric bypass alter the gut-brain communication. Neural Plast..

[27] Geronikolou S.A., Albanopoulos K., Chrousos G., Cokkinos D. (2017). Evaluating the Homeostasis Assessment Model Insulin Resistance and the Cardiac Autonomic System in Bariatric Surgery Patients: A Meta-Analysis. Adv. Exp. Med. Biol..

[28] Windham B.G., Fumagalli S., Ble A., Sollers J.J., Thayer J.F., Najjar S.S., Griswold M.E., Ferrucci L. (2012). The Relationship between Heart Rate Variability and Adiposity Differs for Central and Overall Adiposity. J. Obes..

[29] Sharma R., Hassan C., Chaiban J.T. (2016). Severe Insulin Resistance Improves Immediately After Sleeve Gastrectomy. J. Investig. Med. High Impact Case Rep..

[30] Wu J.M., Yu H.J., Lai H.S., Yang P.J., Lin M.T., Lai F. (2015). Improvement of heart rate variability after decreased insulin resistance after sleeve gastrectomy for morbidly obesity patients. Surg. Obes. Relat. Dis..

[31] Avsar A., Acarturk G., Melek M., Kilit C., Celik A., Onrat E. (2007). Cardiac autonomic function evaluated by the heart rate turbulence method was not changed in obese patients without co-morbidities. J. Korean Med. Sci..

[32] Erdem A., Uenishi M., Matsumoto K., Kucukdurmaz Z., Kato R., Sahin S., Yazici M. (2012). Cardiac autonomic function in metabolic syndrome: A comparison of ethnic Turkish and Japanese patients. J. Interv. Card. Electrophysiol..

[33] Nault I., Nadreau E., Paquet C., Brassard P., Marceau P., Marceau S., Biron S., Hould F., Lebel S., Richard D. (2007). Impact of bariatric surgery--induced weight loss on heart rate variability. Metabolism.

[34] Pabon M.A., Manocha K., Cheung J.W., Lo J.C. (2018). Linking Arrhythmias and Adipocytes: Insights, Mechanisms, and Future Directions. Front Physiol..

